# Comparison of Measurements of Bone Mineral Density in Young and Middle-Aged Adult Women in Relation to Dietary, Anthropometric and Reproductive Variables

**DOI:** 10.3390/nu10111669

**Published:** 2018-11-05

**Authors:** Eloy Méndez-Gallegos, Graciela Caire-Juvera, Humberto Astiazarán-García, Rosa O. Méndez-Estrada

**Affiliations:** Centro de Investigación en Alimentación y Desarrollo (CIAD), Km 0.6 a La Victoria, Hermosillo, Sonora 83304, Mexico; eloy_felipe_48@hotmail.com (E.M.-G.); gcaire@ciad.mx (G.C.-J.); hastiazaran@ciad.mx (H.A.-G.)

**Keywords:** bone mineral density, age at menarche, BMI, nulliparous women, mothers

## Abstract

The objective of this study was to compare current measurements of bone mineral density (BMD) of the lumbar spine (LS), femoral neck (FN), and total femur (TF) regions with initial values recorded 12 years ago in women from Northwest Mexico, and evaluate their correlation with dietary, anthropometric, and reproductive variables. BMD was assessed by Dual-energy X-ray absorptiometry. Participants were grouped as follows: Nulliparous (G1); women who were mothers 12 years ago (G2); and women who were nulliparous 12 years ago, but are now mothers (G3). In all three groups, current LS BMD was higher than initial (*p* ≤ 0.05) and current TF BMD in G2 was higher than initial values (*p* ≤ 0.05). When comparing current FN and TF BMD among the three groups, G2 had higher values than G3 (*p* ≤ 0.05). G2 also showed higher LS BMD than G1 and G3 (*p* = 0.006). Age at menarche was inversely-correlated with FN and TF BMD in G1 (*p* < 0.01), while the body mass index (BMI) correlated positively with all three bone regions in G2 (*p* < 0.05). This study shows that in women without and with children, age at menarche, BMI, and age were factors associated to BMD in healthy subjects in reproductive age.

## 1. Introduction

Bone mass is determined by genetic factors that control values through adolescence and up to the age of approximately 30 years. However, other aspects—some modifiable, others not—are involved in high bone mineral density (BMD) values in adulthood. Factors closely-related to BMD include a balanced diet, regular physical activity (PA), and an adequate body mass index (BMI) [1,2,3,4,5], but some gynecological and reproductive elements have also been associated with the BMD of perimenopausal and postmenopausal women; namely, age at menarche, pregnancy status, and lactation.

Studies have shown that adult women with early onset of menarche (<12 years) have a higher BMD than those with delayed menarche [6], while postmenopausal women who became mothers during adolescence had lower BMD of the lumbar spine (LS), femoral neck (FN) and total femur (TF) than those who were not mothers in that stage [7]. However, these results contrast to those observed during perimenopause, when femoral BMD was higher in women who were adolescent mothers [8].

Regarding the number of pregnancies, Lebel et al. [9] found that the BMD of the LS and TF of adult women was normal regardless of age, number of children, or duration of breastfeeding, but this differs from the inverse association between BMD and number of births reported by Stieglitz et al. [10]. Additionally, when comparing changes in whole-body BMD in adolescent mothers during the first postpartum year to nulliparous adolescents, values were similar despite the observed decrease of BMD at three months postpartum in the adolescent mothers (−0.56%) and the increase in nulliparous women (0.77%). The loss of BMD observed in the adolescent mothers was associated with the number of breast feedings and changes in BMI [11].

In 2012, Mexico ranked first in the proportion of adolescent mothers in OECD countries (Organization for Economic Cooperation and Development) [12]. Statistics compiled by the National Strategy for the Prevention of Adolescent Pregnancy (ENAPEA) indicate that the state of Sonora—in Northwest Mexico—saw an increase in the proportion of adolescent mothers from 15.6 to 18.7% between 2003 and 2012 [13]. 

Against this background, the aim of this study was to compare current measurements of the BMD of the LS, FN, and TF with those made 12 years ago in women from Sonora, and evaluate their correlation with dietary, anthropometric, and reproductive variables. 

## 2. Materials and Methods

### 2.1. Design and Recruitment

Participants were recruited from a 12-month, prospective longitudinal study conducted 12 years ago that assessed variations in the BMD of adolescents in relation to breastfeeding. That work involved 67 women divided into four groups: Adolescent mothers; nulliparous adolescents; adult mothers; and nulliparous adult women [11]. The present study included apparently healthy, non-pregnant women from that original project. Exclusion criteria were: Women under hormone therapy, consumption of dietary supplements, ovarian surgery, and use of medications that affect bone mass, such as those given to treat convulsions (anti-convulsives), respiratory diseases (glucocorticoids), or thyroid problems (thyroid hormones).

Of the total number of participants in the first study [11], seven could not be enrolled in this work because they had changed their residence, two were pregnant, 9 could not be located, and 9 were not interested. Thus, the present sample consists of 40 women, of whom 15 were adolescents (7 mothers, 8 nulliparous) and 25 were adults (17 mothers, 8 nulliparous) 12 years ago. Today, all are adults and of the 16 who were nulliparous, nine now have children. Participants were grouped as follows: Nulliparous (G1, *n* = 9); women who were first-time mothers 12 years ago (G2, *n* = 15); and women who were nulliparous 12 years ago, but are now mothers (G3, *n* = 16). One woman did not undergo the bone densitometry analyses for reasons unrelated to the study (G2, *n* = 14), but all questionnaires and anthropometric measurements were applied in all cases. Both the earlier and current measurements were taken using the same methodology.

A written informed consent from each participant was obtained. The study protocol and consent forms were approved by the Ethics Committee of the Centro de Investigación en Alimentación y Desarrollo and was conducted in accordance with the Helsinki Declaration. 

### 2.2. Questionnaires

A questionnaire was applied to register the number of children and duration of breastfeeding by the participating mothers. To assess the women’s socioeconomic level, we used a survey prepared by the Mexican Association of Research Agencies [14] that includes questions on marital status, parents’ educational level and whether the household has an automobile, among others. After analyzing the responses, socioeconomic levels were assigned as follows: A/B, C, D, and E, with A/B representing the highest standard of living and E the lowest quality of life or wellbeing.

### 2.3. Anthropometric Evaluation

For the original (12 years ago) and current anthropometric evaluations, the body weight (kg) and height (cm) of each woman were measured following standardized procedures while they were wearing light clothing, without shoes. An electronic scale AND HV-200 KGL (A&D Co., Seoul, South Korea,) and 20–205 cm SECA stadiometer, model 213 (GmbH & Co, KG, Hamburg, Germany) were used. BMI was calculated using the Quetelet formula, which divides weight (kg) by height (m^2^). The cutoff points were: <18.5, underweight; 18.5–24.9, normal; 25–29.9, overweight; ≥30 obese; 30–34.9 obesity type I; 35–39.9, obesity type II; and ≥40, obesity type III [15]. 

### 2.4. Dietary Evaluation

Dietary intake was estimated by a food consumption frequency survey previously evaluated and validated for the Mexican population [16] and slightly modified for this study. This dietary tool was applied to estimate food consumption during the previous year. It included 104 foods grouped in 11 categories: Dairy products, eggs, meat, sausages, vegetables, legumes, cereals, candies, drinks, oils and fats, and regional foods. The frequency of consumption of these items was evaluated on a daily, weekly, or monthly basis. A trained professional interviewer applied the survey and the data obtained were analyzed using the Nutritional Habits and Nutrients Intake Evaluation System (SNUT) [17]. 

### 2.5. Physical Activity

Physical activity (PA) was also estimated using a questionnaire, which records the time that participants spent on different activities during the week; for example, how much time they spend cleaning the home, walking, running, playing volleyball or cycling, among other forms of exercise. Based on these data, PA was calculated in terms of multiples of basal metabolism (mMB). Depending on their level of activity, participants were classified as sedentary (values between 1.40–1.69 mMB), moderately active (1.70–1.99 mMB), or intensely active (2–2.4 mMB) [18].

### 2.6. Evaluation of Bone Mineral Density

The values obtained for weight, height, age and sex were entered into the program of the Lunar dual X-ray densitometry DPX-MD+ using software 5.0 (GE, Milwaukee, WI, USA). Each woman was placed in the position indicated in the manufacturer’s protocol and measurements of the femoral neck (FN), total femur (TF), and lumbar spine (LS) were taken.

### 2.7. Statistical Analysis

To compare the BMD, dietary and reproductive information from the current study to the results from 12 years ago, we used the paired *t*-test. The inclusion of covariates was based on the results of a stepwise regression search, in which the probability for entry was set at 0.2 (number of children, BMI, age at menarche and years of age, protein, phosphorus, and fat intake). A Pearson correlation analyses was performed to determine the relationship between each variable analyzed and BMD. Spearman’s correlation was used for non-parametric data. Differences in BMD among the three groups were evaluated using an ANCOVA analyses, and the Tukey Kramer’s test was applied to prove differences between groups. For the regression analyses we considered the moderate variance inflation factors (VIF) under 10, to avoid collinearity. Normality of residuals, homoscedasticity, and linearity were tested to assure a valid regression model. Data analyses were performed using the NCSS v7 statistical package with statistical significance set at *p* ≤ 0.05. 

## 3. Results

Table 1 presents the following data: Age, age at menarche, number of children, BMI, duration of breastfeeding, and protein, phosphorus, calcium and fat intake. Twelve years ago, the age range of participants was 13–32 years with approximately 60% ≤20 and only one >30. In the current study, subjects’ ages were as follows: G1, 24–38 (56%, >30); G2, 26–37 (50%, >30); and G3, 25–43 (75%, >30). BMI increased from 12 years ago to current values in all three groups. The proportion of women with normal weight (87.2%), overweight (7.7%) and low weight (5.1%) 12 years ago changed to 53.8%, 35.9% and 0%, respectively. In addition, 5.1% of the women in the current study presented obesity type I, and the same percentage presented obesity type II.

In both phases of the study, protein and phosphorus intake exceeded the recommended daily allowances (46 g/day of protein for women over 14; 700 mg/day of phosphorus for women over 19) [19,20], but calcium intake in all groups was below the recommended amount (1000 mg/day) [20]. The target Ca:P ratio of 1:1 was not attained by any group in both period. The fat intake was higher in the beginning compared with the actual for G1, but the consumption in the other groups remained similar. The mean fat intake (35.7%) was in the highest limit of the recommendation for adults (20–35% of energy); however, 62.5% of the women in this study had a high fat intake [19]. None of the nutrients showed associations with the BMD of the measured regions. In relation to PA, all subjects were classified as sedentary, while in terms of socioeconomic level, 25% were in the highest brackets, 62.5% in the middle, and 12.5% in the lowest.

Table 2 shows the LS, FN and TF BMD of the 3 groups. The comparison of initial BMD values, from the three osseous regions, showed no significant differences among groups (p^1^, *p* > 0.05). The comparison of current BMD values, showed significant differences among groups (p^2^, *p* > 0.05) for LS (*p* = 0.006), FN (*p* = 0.02) and TF (*p* = 0.03). The comparison between initial and current values, showed that current LS BMD was higher than the initial measurements for G1 (*p* = 0.004), G2 (*p* = 0.001) and G3 (*p* = 0.050. Likewise, TF BMD was higher in the current measurements, but only in G2 (*p* = 0.003).

The correlation results between current LS, TF, and FN BMD with the anthropometric, dietary and reproductive variables showed that age at menarche was inversely-correlated with FN and TF BMD in the nulliparous women (G1) (r = −0.8, *p* = 0.001; r = −0.8 *p* = 0.006, respectively). BMI was positively−correlated with LS, FN, and TF BMD in those who were mothers 12 years ago (G2) (r = 0.6, *p* = 0.01; r = 0.6, *p* = 0.008; and r = 0.7, *p* = 0.004, respectively). G3 subjects did not show correlations with any variable (Table 3).

In the regression analyses, FN BMD was the only variable associated to the independent variables. By univariate regression, FN BMD was negatively associated to age at menarche, but only for G1. By multiple regression, the FN BMD of all the study women together (*n* = 39) was negatively associated to years of age before and after adjusting for the independent variables. Additionally, FN BMD was negatively associated to age at menarche, while BMI was positively associated, after adjusting for different variables (Table 4).

## 4. Discussion

We measured the LS, FN and TF BMD of adult women (mothers and nulliparous) who had been evaluated 12 years ago (initial measurement; adolescents and adults, mothers and nulliparous women) to: (1) Compare the BMD between the two sets of measurements; and (2) identify the factors related to the current BMD values. Among potential factors, studies have posited that age [21], BMI [22], age at menarche [23], reproductive variables (number of children and postmenopause status) [24,25], and diet (calcium, protein, and phosphorus intake) [26,27,28], show associations with BMD in women in the pre and postmenopausal stages.

The age at which the maximum value of bone mass is reached (i.e., peak bone mass, PBM) depends on racial and genetic factors, among others [29,30]. In Italian women, maximum LS BMD is reached between 20–49 years [31], in Spanish women at age 30–39 [32], in Canadian women, between 33–40 years [33], and in Vietnamese women it is estimated at 25–29 years [34]. Meanwhile, maximum FN BMD occurs between 20–29 years of age in Italian and Spanish women, 16–19 years in Canadian women, and 24–29 years in Vietnamese women. There is no information on the age at which Mexican women reach PBM, but the ranges reported in other countries justify considering that the age of the women in this study corresponds to the stage called bone mass conservation. 

Regarding the association between age at menarche and BMD in premenopausal adult women, a relation between early menarche and high BMD has been documented [35]. This has been attributed to more years of reproductive life [36] and, therefore, longer exposure to estrogen [6]. In a previous study conducted in the same geographical region as the present one, the average age at menarche was 12.06 years [37], while in two cities in Mexico (Mexico City and Xalapa [38]), it was found to be 11.40 and 11.34 years, respectively. In the present study, age at menarche of all participants ranged from 10–15 years and was inversely-correlated to current FN and TF BMD in the nulliparous women.

In addition, after adjusting for age at menarche and BMI, current FN and TF BMD were higher in the women who were mothers 12 years ago (G2) than in those who were nulliparous 12 years ago but are now mothers (G3). According to Tryniszewski et al. [39], adult women, young and over 50 with BMI > 25 have higher FN BMD values than those with BMI < 25. Additionally, several studies have reported that obesity during childhood, adolescence, and adulthood increases bone mass [4,40]. In adults, body weight is one of the most important environmental factors that influence the gain of bone mass [30]. Therefore, it has been associated with increased vertebral bone density and increased whole-body bone dimensions and mass during childhood and adolescence [40]. In adolescents under natal hormonal control (*n* = 245), and in a reference group (*n* = 188), Bonny et al. [41] reported a positive correlation of body weight with FN and LS BMD, as well as changes in body weight with FN BMD at 12 and 24 months after the study began. In elderly Spanish women with overweightness/obesity, Gómez et al. [42] reported higher BMC and BMD than in their non-overweight peers in sub-total body (whole body-head), LS, hip, and FN, though after adjusting for lean mass some of those associations ceased to be significant. Tanaka et al. [43], meanwhile, reported a higher risk of fractures in the femurs of Japanese women with low or normal weight, and in LS in women with obesity.

Regarding the mechanisms involved in the BMI-BMD-fracture hazard association, Ishii et al. [44] point out that although BMD increases with greater skeletal loading, the difference in BMD is insufficient to compensate for the increase in the fall impact forces in obese individuals. However, considering that fat mass is a source of inflammatory cytokines, including IL-6 and TNF-alfa, it is to be expected that increasing the amount of fat mass (obesity) will contribute to the development of a chronic inflammatory state that can lead to inflammatory bone loss [45,46].

In the present study, BMI positively influenced LS, NF and TF BMD in the women who were mothers 12 years ago. It is important to note that this group showed the highest rate of overweightness and obesity of all study participants, with levels of 50% and 14.3%, respectively. Despite their positive effect on bone mass, these values entail serious health risks.

The women in the present study reported sedentary physical activity (PA), but this was not associated with the BMD of the bone regions measured. The association between PA and BMD has been evaluated in Irish women and men, where it proved to correlate positively with LS BMD in men, while work-related activities and other non-sports activities were not associated with BMD in women [47]. In Sweden, higher levels of LS and hip BMD were associated with the high recreational activity level of 25-year-old women [48], while in a U.S. population sitting was negatively-associated with TF BMD in women aged 23 to over 90 [49]. In postmenopausal women in Canada, increasing the number of daily steps and doing tasks of daily living prevents a decrease in BMD [50].

There was a significant inverse association between FN BMD and age at menarche when adjusted with BMI and years of age for the total of women; while there was an inverse association with age at menarche and FN BMD in G1 by simple regression. Ito el al. [35] reported an inverse correlation between age at menarche by quantitative computed tomography and in LS BMD in pre and post-menopausal women. Additionally, a positive correlation between body weight and FN, and LS BMD was reported by Bonny et al. [41]. Regarding age and its negative association with BMD, there is an England study with elderly men and women in which there were inverse associations with age and FN and Wards triangle BMD in both men and women. Additionally, they reported that after the age of 65, BMD decline continued in men and women [21].

In terms of inadequate calcium intake, studies have shown low LS and FN BMD values in women who consume less than 400 mg daily, whereas in men who consume more than 1200 mg daily this was positively-associated with FN and TF BMD [51]. In Finland, Kemi et al. [52] reported that four groups of women aged 20–28 received supplements with different amounts of phosphorus or placebo and consumed 250 mg of calcium. The group that consumed the highest amount of phosphorus (1500 mg) showed a decrease in serum calcium. In addition, there was a decrease in a marker of bone formation with an increase in a marker of bone resorption, though only in the group with the highest phosphorus intake. In their study, Lee and Cho [53] reported that in women aged 13–99, high consumption of phosphorus does not negatively affect bone metabolism when calcium intake is adequate. 

In young women (18.47–26 years), nutrition (calcium intake, calcium/protein ratios) and physical activity may have a positive effect on the rate of gain in spinal BMD, but the gain in bone mass ends before age 30 [54]. The average age of the women in our study was approximately 31 years. While their calcium consumption did not meet the recommended daily allowance, LS BMD showed higher values in the current measurements than in the initial ones in all three groups. In contrast, current BMD in the femur increased only in the women who were mothers 12 years ago (G2).

Different studies have shown that dietary fat intake affects BMD depending on the consumed amount of fat. Kwon et al. [55] reported that in women with medium fat energy intake, BMD was higher than in those with a low and high fat energy intake. Parhami et al. [56] in a study with animals, considered that a possible mechanism that could explain the decrease of BMD with a high fat diet is an inhibition of osteoblastic differentiation and bone formation. Nevertheless, McTiernan et al. [57] in an intervention study observed a hip BMD lower in women who received a dietary modification (increased amount of fruit, vegetable, grain, and a low fat diet), than the group who did not received any dietary changes. In general, our results showed that 62.5% of the women had a high fat intake; however, we did not find any association between fat intake and BMD in any measured region.

Regarding the impact of pregnancy during adolescence on BMD, Cho et al. [7] reported lower BMD values in TF, FN, and LS in postmenopausal women who had a history of pregnancy during adolescence, compared with women who were not teenage mothers. However, Yüce et al. [8] reported that TF BMD in postmenopausal women was higher in those with a history of pregnancy during adolescence than in those who had children at a later age. In our study, LS BMD increased in all three groups. After adjusting for the number of children, G2 showed higher values than G3, regardless of whether the women were adolescents or young adults, with or without children at the time of the initial measurements. Additionally, a possible protective effect of motherhood on BMD was observed, given that TF BMD increased in the women who were mothers 12 years ago, similar to that reported by Yüce in postmenopausal women [8].

## 5. Conclusions

This study shows that in women without and with children, respectively, age at menarche, and BMI were factors related to BMD in healthy subjects in reproductive age. The highest proportion of women with unhealthy BMI corresponded to those who became mothers at an earlier age and contributed to their higher BMD in all three bone regions studied, compared to the nulliparous women and those who had children at a later age. It is necessary to correct low calcium intake and the level of physical activity to help maintain BMD and reduce the risk of bone loss in women in the premenopausal age range. 

## Figures and Tables

**Table 1:** Age, reproductive factors and BMI plus protein, phosphorus and calcium intake among participants.

Variable	G1 (*n* = 9)		G2 (*n* = 15)		G3 (*n* = 16)	
	Initial	Current	Initial	Current	Initial	Current
Age (years)	18 ± 4.7	29 ± 4.6	19 ± 3.4	30 ± 3.5	22 ± 5.4	33 ± 5.3
Age at menarche (years)	11.7 ± 1.3	11.7 ± 1.3	13.1 ± 0.8	13.1 ± 0.8	12.1 ± 1.1	12.1 ± 1.1
Number of children	-	-	1	1–4	-	1–4
BMI	20.4 ± 1.7	23.1 ± 3.8 *	22.6 ± 2.1	25.9 ± 4.02 *^,†^	21.5 ± 2.4	25.9 ± 5.2 *^,†^
Low weight	11.2%	0%	7.1%	7.1%	18.7%	0%
Normal	88.8%	77.7%	78.5%	28.5%	75%	56.2%
Overweight	0%	22.3%	14.4%	50%	6.3%	31.2%
Obesity I	0%	0%	0%	14.4%	0%	0%
Obesity II	0%	0%	0%	0%	0%	12.6%
Breastfeeding (months)	-	-	6.3 ± 5.12	6.3 ± 3.7	-	6.5 ± 4.7
Protein (g/day)	90.41 ± 22.7	83.78 ± 43.7	92.1 ± 40.9	81.3 ± 31.1	79 ± 25.7	77.9 ± 37.7
Phosphorus (mg/day)	1394.8 ± 392	1320.3 ± 765	1423.3 ± 656	1114.2 ± 364	1237.4 ± 423	1162.9 ± 160
Calcium (mg/day)	927.16 ± 435	608.5 ± 284 *	947.75 ± 511	572.76 ± 220 *	781.3 ± 309	554.8 ± 64.1 *
Fat (mg/day)	105.07 ± 14.64	67.56 ± 20.71 *	103.33 ± 50.99	75.65 ± 35.87	91.10 ± 30.92	86 ± 51.58

Values are mean ± SD. Paired *t* test for normal variables. ^†^ Wilcoxon test for non-parametric variables. G1: nulliparous women; G2: mothers 12 years ago; G3: nulliparous 12 years ago but now mothers. BMI: body mass index. * Significant differences at *p* ≤ 0.05.

**Table 2:** Comparison of initial and current LS, FN, and TF BMD values between groups of participants.

Variable	Initial				Current			
	G1	G2	G3	p^1^	G1	G2	G3	p^2^
LS BMD	1.10 ± 0.03	1.12 ± 0.02	1.08 ± 0.02	0.58	1.15 ± 0.04 ^b^	1.27 ± 0.03 ^a^	1.13 ± 0.03 ^b^	0.006
FN BMD	1.04 ± 0.03	1.02 ± 0.03	0.99 ± 0.02	0.55	1.03 ± 0.03 ^a,b^	1.07 ± 0.02 ^a^	0.96 ± 0.02 ^b^	0.02
TF BMD	1.02 ±0.03	1.00 ± 0.02	1.00 ± 0.02	0.81	1.03 ± 0.04 ^a,b^	1.07 ± 0.03 ^a^	0.96 ± 0.03 ^b^	0.03

Values are means ± SE. G1: nulliparous women (*n* = 9); G2: mothers 12 years ago (*n* = 14); G3: nulliparous 12 years ago but now mothers (*n* = 16). LS: lumbar spine; FN: femoral neck; TF: total femur. Means in a row with different superscript letters are significantly different (comparison of initial measurements among the three groups and the same comparison with current measurements). p^1^, statistical significance with the initial BMD measurements among the 3 groups. p^2^, statistical significance with current BMD comparison among the 3 groups. Paired *t* test between current study and previous study in each group. General Linear Models with Tukey’s test; *p* ≤ 0.05.

**Table 3:** Correlation between age at menarche and BMI with LS, TF, and FN BMD.

Variable	Bone Region	G1		G2		G3	
		r	*p*	r	*p*	r	*p*
Age at menarche	FN	−0.8	0.001 *	−0.1	0.5	−0.3	0.1 ^†^
Age at menarche	TF	−0.8	0.006 *	−0.3	0.2	−0.1	0.4 ^†^
BMI	LS	−0.2	0.50	0.6	0.01 *	0.1	0.4 ^†^
BMI	FN	−0.3	0.40	0.6	0.008 *	0.1	0.5 ^†^
BMI	TF	−0.02	0.91	0.7	0.004 *	0.4	0.1 ^†^

LS: lumbar spine; FN: femoral neck; TF: total femur. G1: nulliparous women; G2: mothers 12 years ago; G3: nulliparous 12 years ago but now mothers. ^†^ Spearman, non-parametric variables; * Correlations are significant at *p* ≤ 0.05.

**Table 4:** Regression analyses of femoral neck BMD, age at menarche, BMI, and years of age in the total population and G1.

Group (*n*)	Region Measured	Variable	β Crude	*p*	β Adjusted	*p*
G1 (9)	FN	Age at menarche (years)	−0.0755 ^a^	0.0016 *		
Total (39)	FN	Age at menarche (years)	−0.0307	0.0568	−0.0346 ^b^	0.0145 *
		BMI (kg/m^2^)	0.0050	0.2439	0.0098 ^c^	0.0125 *
		Years of age	−0.0091	0.0218 *	−0.0126 ^d^	0.0011 *

^a^ Simple linear regression; ^b^ adjusted by BMI and years of age; ^c^ adjusted by age at menarche and years of age; ^d^ adjusted by age at menarche and BMI; G1: nulliparous women; * significant association at *p* ≤ 0.05. FN: femoral neck; BMI: body mass index.
